# An artificial intelligence-enhanced coaching mode

**DOI:** 10.1097/JS9.0000000000002713

**Published:** 2025-06-23

**Authors:** Ke Cheng, Shangdi Wu, Bing Peng, Xin Wang

**Affiliations:** a Department of General Surgery, Division of Pancreatic Surgery, West China Hospital of Sichuan University, Chengdu, China; b School of Medicine West China, West China Hospital of Sichuan University, Chengdu, China

**Keywords:** surgical coaching, artificial intelligence, laparoscopic pancreatoduodenectomy, smart coach

## Abstract

Surgical coaching has emerged as an innovative educational strategy designed to enhance both the technical and nontechnical competencies of surgeons through structured, individualized feedback. As minimally invasive surgical techniques continue to proliferate, video-based coaching has proven effective for skill refinement. However, its broader implementation remains limited due to a shortage of expert coaches and the labor-intensive nature of video review. Advances in artificial intelligence (AI), particularly in the field of computer vision (CV), present promising opportunities to optimize surgical coaching by automating video analysis and enabling scalable, data-driven feedback mechanisms. This study introduces SmartCoach, an AI-assisted surgical coaching program designed to support laparoscopic pancreatoduodenectomy – a technically demanding procedure typically reserved for highly experienced surgeons. The program integrates an intelligent visualization system and structured postoperative debriefings to identify key performance issues and foster targeted improvement strategies. Preliminary survey data revealed limited awareness among participating surgeons regarding surgical coaching principles and the role of AI in surgical education. While most reported frequent use of operative videos for learning, they cited the lack of expert feedback and inefficiency as major barriers. The AI-driven coaching model seeks to address these challenges by providing real-time intraoperative assessments, automated identification of surgical steps, and enhanced scalability facilitated by 5G-enabled communication technologies. Despite its promise, the implementation of AI-based coaching faces ethical, logistical, and cultural obstacles, including data privacy concerns and resistance to change among experienced surgeons. Nonetheless, the integration of AI into surgical coaching represents a transformative step toward improving operative performance, surgeon well-being, and patient outcomes, particularly in highly complex procedures where expert support is often limited.

## Introduction

Surgical coaching represents an innovative educational paradigm designed to improve surgeons’ technical competencies and clinical decision-making skills through structured, personalized feedback^[1]^. With the widespread adoption of minimally invasive techniques, video-based surgical coaching – encompassing video review, self-debriefing, refinement of intraoperative decision-making, and analysis of counterexamples such as unnecessary movements – has emerged as a valuable method for skill development^[2]^. Studies showed its advantages in improving surgeons’ technical and nontechnical skills, as well as overall well-being^[3]^. However, the broader implementation of surgical coaching is hindered by a limited availability of trained coaches and the time-intensive nature of manual video review. The rapid development of artificial intelligence (AI), particularly in the domain of computer vision, has presented an opportunity for the efficient promotion of video-based surgical coaching. This research letter explores the utility of an AI-based surgical coaching system in enhancing surgical education. The study is compliant with the TITAN Guidelines 2025^[4]^.

**HIGHLIGHTS**

- Artificial intelligence (AI)-driven video analysis enhances surgical coaching by streamlining procedural identification and performance evaluation, reducing reliance on manual expert review.
- The SmartCoach program demonstrates the potential of AI-assisted coaching to enhance trainee performance in complex procedures like laparoscopic pancreatoduodenectomy.
- AI-based coaching models, when integrated with technologies such as 5G, offer scalable and remote solutions for structured surgical education.

## The crucial role of AI in surgical video coaching and reviewing

AI Offers several advantages in the context of surgical coaching, including advanced data analysis capabilities, personalized learning design, intraoperative decision support, and the ability to overcome temporal and spatial limitations. For example, AI models have demonstrated the capacity to automatically identify surgical steps, instruments, and the Critical View of Safety (CVS) score during laparoscopic cholecystectomy (LC)^[5]^. Traditional surgical coaching is mostly undertaken by clinical experts who are burdened with heavy clinical, administrative, and research commitments. For complex procedures characterized by multiple surgical steps and significant anatomical variability, video review becomes particularly time-consuming, further hindering the scalability and routine implementation of video-based coaching programs. AI facilitates the efficient analysis of surgical procedures by enabling the rapid recognition of surgical steps, intraoperative events, and technical maneuvers^[6]^. Moreover, AI-generated objective data can be promptly cross-referenced with multicenter surgical databases, allowing for comprehensive performance assessment and the development of tailored improvement strategies. Certain AI-based analytical tools also offer real-time intraoperative decision support – for example, automated CVS scoring during LC. In addition, the development of information transmission technologies, particularly 5G networks, eliminates temporal and spatial constraints for surgical coaching, substantially improving its scalability and widespread applicability.

## AI-coaching program in laparoscopic pancreatoduodenectomy

There are numerous evidence to support the effectiveness of video-based surgical coaching in technical and nontechnical skill refinement. Khan *et al*^[7]^ demonstrated an AI-assisted surgical steps identification coaching program in pituitary surgeries and participants showed improvement of surgical performance. Our previous study constructed an AI-enhanced model SurgSmart to automatically output surgery report including surgical steps length, critical division action, CVS score information in LC. Integration of this AI model into our coaching program resulted in significant improvements in both operative performance and patient safety among novice LC surgeons^[8]^. Given that senior surgeons typically manage more complex cases yet face greater difficulties in obtaining external assistance compared to junior colleagues, AI-based coaching systems may offer particularly high value in advanced surgical procedures. AI-enhanced surgical coaching in the context of complex surgical procedures remains largely unexplored at present.

**Figure 1.**
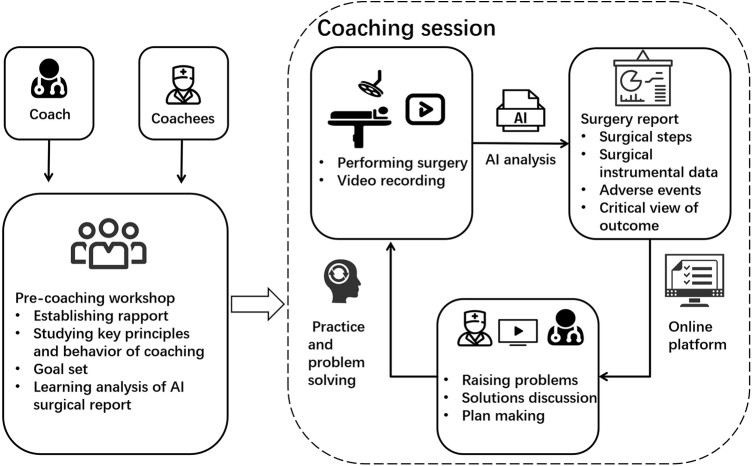
Artificial intelligence (AI) assisted surgical coaching workflow.

To further explore its influence in complex surgeries, we first performed an AI-coaching program in laparoscopic pancreatoduodenectomy (LPD). Based on established coaching principles and validated models, we designed the workflow of the SmartCoach program for LPD coaching (Fig. 1). Prior to participation, both coaches and coachees were required to learn the core principles of surgical coaching. Following each procedure, a 30-minute online coaching session was conducted. These sessions involved in-depth, structured discussions between the coach and coachee. Using the surgical report and an intelligent visualization system, the coach facilitated efficient debriefing to identify key performance issues. Constructive feedback was provided accordingly, and targeted improvement strategies were collaboratively developed to guide the coachees’ approach to the next procedure.

To assess participants’ baseline understanding and perceptions, we administered a questionnaire survey. The results highlighted several key deficiencies in routine surgical learning practices. First, although all participants were senior surgeons with extensive operative experience, 64.7% reported no prior exposure to the concept of surgical coaching, and most demonstrated limited awareness of AI applications in surgical education (Table 1). Second, although most of them often review and learn through surgical videos, they believed that the lack of expert-guided review and unsatisfied efficiency were the main obstacles to their learning. Third, the survey also revealed indicators of well-being concerns among some participants (Table 2). The full questionnaire and study details can be found in the Supplemental Digital Content, available at: http://links.lww.com/JS9/E432.

**Table 1:** Demographics and Questionnaire results of AI coaching program

Characteristics	Number of doctors	Percentage
Surgeons title		
Chief	5	29.41%
Vice chief	11	64.71%
Attending	1	5.88%
LPD surgical experience		
Less than 5 cases	4	23.53%
5–10 cases	3	17.65%
10–20 cases	2	11.76%
More than 20 cases	8	47.06%
Laparoscopic surgery experience		
Less than 5 years	1	5.88%
5–10 years	5	29.41%
10–15 years	3	17.65%
More than 15 years	8	47.06%
Way to learn surgery		
Surgical videos	15	88.24%
Academic conferences	12	70.59%
Surgical training courses	9	52.94%
Self-learning	4	23.53%
Awareness of the coaching model in surgical training		
Yes	3	17.65%
No	11	64.71%
Unsure	3	17.65%
Awareness of AI applications in surgical education		
Never	1	5.88%
Rarely	9	52.94%
Occasionally	4	23.53%
Frequently	3	17.65%
Do you frequently conduct video reviews of surgical procedures		
Rarely	1	5.88%
Occasionally	8	47.06%
Frequently	8	47.06%
Preference for reviewing own or others’ videos		
Own videos	11	64.71%
Others’ videos	4	23.53%
No preference	2	11.76%
Preferred type of video for review		
Surgeries subjectively needing improvement	7	41.18%
Surgeries with adverse events or complications	5	29.41%
Standardized surgeries by experts	5	29.41%
Biggest challenge in current surgical video review learning		
Lack of expert guidance	10	58.82%
Too time-consuming	3	17.65%
Lack of surgical video resources	2	11.76%
Lack of effective review methods and tools	2	11.76%
Which surgical steps are your primary concerns in LPD? (multiple selections)		
Duodenal mobilization	6	35.29%
Resection of the pancreatic uncinate process	12	70.59%
Pancreaticojejunostomy	12	70.59%
Lymph node dissection	7	41.18%
Most desired learning techniques for LPD		
Traction and exposure techniques	3	17.65%
Hemorrhage management techniques	7	41.18%
Digestive tract anastomosis techniques	4	23.53%
Lymph node dissection techniques	3	17.65%

LPD, laparoscopic pancreatoduodenectomy.

**Table 2:** Well-being results of AI coaching program

Well-being	Number of doctors	Percentage
Reduced empathy toward patients		
Extremely true	0	0.00%
Very true	0	0.00%
True	3	17.65%
Partly true	3	17.65%
Not at all	11	64.71%
Lack of work motivation		
Extremely true	0	0.00%
Very true	0	0.00%
True	2	11.76%
Partly true	4	23.53%
Not at all	11	64.71%
Physical exhaustion		
Extremely true	0	0.00%
Very true	0	0.00%
True	2	11.76%
Partly true	10	58.82%
Not at all	5	29.41%
I experience a sense of dread when anticipating obligatory work tasks		
Extremely true	0	0.00%
Very true	2	11.76%
True	2	11.76%
Partly true	4	23.53%
Not at all	8	47.06%
I’m satisfied with my job		
Extremely true	2	11.76%
Very true	6	35.29%
True	5	29.41%
Partly true	3	17.65%
Not at all	1	5.88%
I find joy in my work		
Extremely true	0	0.00%
Very true	5	29.41%
True	4	23.53%
Partly true	6	35.29%
Not at all true	1	5.88%
My work feels highly valuable		
Extremely true	4	23.53%
Very true	4	23.53%
True	4	23.53%
Partly true	4	23.53%
Not at all true	1	5.88%
I contribute professionally at my best capacity (patient care/teaching/research)		
Extremely true	3	17.65%
Very true	5	29.41%
True	4	23.53%
Partly true	5	29.41%
Not at all true	0	0.00%

## The opportunities and challenges of AI-coaching mode

The AI-based surgical coaching system is expected to address these issues above. In this study, we found that senior surgeons have a need to improve their surgical skills, but the current traditional teaching model is time-consuming and inefficient. The AI-coaching model has demonstrated utility in basic surgeries such as LC and pituitary surgery, but its application in complex procedures remains unexplored. Given the scarcity of experts capable of mentoring advanced surgeries, AI-coaching may represent a more effective training modality for enhancing the surgical proficiency of senior surgeons. However, there still many challenges faced. We share a vision that we must ensure the security of video data, patient confidentiality, and consent from operating surgeons. Some surgeons may refuse to share their videos and to self-debrief because of dignity problem and data security. Studies showed that some surgeons had performed a large number of cases but the quality of surgeries was still not so satisfied^[5]^. Another significant challenge is the current lack of formal coaching programs to train professional coaches in the principles and techniques of surgical coaching.

## Conclusion

Our investigations have demonstrated the necessity of AI-assisted surgical coaching in complex procedures. The application of AI-coaching mode offers transformative opportunities to improve operative performance and well-being in surgeons. Currently, both surgical practitioners and hospital administrators now recognize the critical value of video-derived surgical data for training and quality improvement. As legal frameworks evolve and professional attitudes shift, routine coaching is emerging as an effective strategy to enhance both technical and nontechnical surgical skills, ultimately improving patient outcomes and procedural quality.

## Data Availability

Not applicable.
